# EFFECTIVENESS AND ADVERSE EFFECTS OF IMMERSIVE TECHNOLOGIES USED FOR REHABILITATION OF PATIENTS WITH NON-SPECIFIC NECK PAIN: A SYSTEMATIC REVIEW

**DOI:** 10.2340/jrm.v57.42108

**Published:** 2025-01-31

**Authors:** Joanna S. KOSTKA, Agnieszka ZAWADZKA-FABIJAN, Dariusz DZIAŁA, Bogumila BRUC, Magdalena PRUSZYŃSKA, Gabriela FIGAS, Rory J. O’CONNOR, Antti MALMIVAARA, Jolanta E. KUJAWA

**Affiliations:** 1 Department of Gerontology, Medical University of Lodz, Lodz, Poland; 2 Clinic of Medical Rehabilitation, Medical University of Lodz, Lodz, Poland; 3 Information and Library Center, Medical University of Lodz, Lodz, Poland; 4 Department of Internal Diseases, Rehabilitation and Physical Medicine, Medical University of Lodz, Lodz, Poland; 5 Academic Department of Rehabilitation Medicine, Leeds Institute of Rheumatic and Musculoskeletal Medicine, University of Leeds, Leeds, UK; 6 National Demonstration Centre in Rehabilitation, Chapel Allerton Hospital, Leeds, UK; 7 National Institute for Health and Welfare, Helsinki, Finland; 8 Orton Orthopaedic Hospital, Helsinki, Finland

**Keywords:** neck pain, physical therapy modalities, rehabilitation, virtual reality, gamification

## Abstract

**Objective:**

The aim of this study is to evaluate the effectiveness of immersive technologies in the rehabilitation of patients with non-specific neck pain and identify any potential side effects associated with their use.

**Design:**

Systematic review.

**Subjects/Patients:**

Individuals with non-specific neck pain.

**Methods:**

A systematic literature search of randomized controlled trials was conducted using Medline (PubMed), Embase (Ovid), Scopus, Cochrane Database of Systematic Reviews, WHO, Pedro, and ClinicalTrials.gov. Risk of bias was assessed with Cochrane Risk of Bias tool.

**Results:**

Five studies with a total of 203 participants (129 women, 74 men) were included in the review. In most studies, both the virtual reality (VR) and control groups demonstrated improvement in pain, functioning related to neck pain, and range of motion. Two cases found the virtual reality group to demonstrate greater improvements in pain and range of motion (for some movements), but not in function. The studies analysed lack much information regarding the applicability of VR therapy.

**Conclusion:**

The data are promising and suggest that VR therapy may have benefits in the rehabilitation of patients with non-specific neck pain. Data on the safety of therapy and adverse events are insufficient to draw any conclusions.

## LAY ABSTRACT

Non-specific neck pain syndrome is a widespread problem in the adult population, requiring frequent preventive and rehabilitation interventions. The preferred treatment involves a combination of manual therapy, exercise, and education. New possibilities lie in the use of virtual reality. This study reviews the evidence to assess the feasibility, effectiveness, clinical applicability, and potential adverse effects of using these interventions. The data are promising and may indicate that virtual reality may be as beneficial in the rehabilitation of patients with non-specific neck pain as current treatments. However, the studies are very different from each other and the lack of clinical data on safety and adverse events limit our ability to recommend it for clinical practice. There is considerable need for further evidence on the effectiveness and harm of VR-based therapy for neck pain.

As the people living in our society age, and lifestyle factors that favour the incidence of musculoskeletal conditions become more prevalent, the demand for rehabilitation for these conditions will grow. Cervical spine disorders, including neck pain, are associated with increased disability and economic costs, requiring rehabilitation and preventive interventions (1). The Global Burden of Disease Study estimates the prevalence of neck pain to be 3,551 people affected per 100,000 population in 2017, with people in Norway, Finland, and Denmark being most affected (2). Neck pain is a broad concept. It may be related to a specific disease or injury, or it may be non-specific neck pain, when a pathological process cannot be identified as the cause of the pain (3).

Guidelines for the treatment of patients with neck pain mainly recommend non-pharmacological interventions combining manual therapy, exercise, and education (4, 5). However, there is a need for more effective strategies that offer greater acceptability to patients, ease of delivery and cost effectiveness. The COVID-19 pandemic generated greater interest in treatments that were safe for patients and healthcare professionals, with one option being the use of virtual reality (VR) (6). Fully immersive VR systems give the impression of presence, the ability to explore and interact with an artificially generated virtual environment with isolation from the real world through the use of head-mounted displays (7). VR systems have been studied in the rehabilitation of stroke patients (8), orthopaedic patients (9), children (10), cardiac patients (11), and older adults (7). VR-based rehabilitation can also be used in the home environment (12). It is important to determine whether such therapy is effective, safe, and well tolerated, and this is especially important in the case of neck pain. While virtual rehabilitation has potential advantages, it requires additional equipment, such as glasses and head-mounted displays, which may have negative effects in this group of patients. Studies suggest that using a head-mounted display may result in a change in head and neck posture, consequently leading to greater stress on the musculoskeletal system (13). In addition, people with neck pain may be more likely to experience dizziness, nausea, visual disturbances, and other symptoms (14) similar to cybersickness (15), which may also present a barrier to the use of VR-based therapy. To enable generalization of results from systematic reviews and their applicability to clinical practice, clear reporting of results, taking into account factors that may influence the outcome of therapy, is essential.

The success of VR therapy may depend on factors such as age, sex, environmental factors, initial characteristics of the condition, and therapy parameters. For example, older patients may be wary of using new technologies, including VR, and its use may give rise to additional psychological distress, which may reduce its effectiveness (16, 17). Therefore, there is a need to better understand the opportunities afforded by VR technology, and the patients to whom it is best suited. The use of the benchmarking method (BM) is recommended to assess the ability of systematic reviews to capture important elements in randomized controlled trials (RCTs) (18).

Previous reviews of the effectiveness of treatment of neck pain with VR technologies (19–23) were based on a heterogeneous group of studies and lacked sufficient data that would allow clinicians to directly translate the results into practice. These have included pain occurring throughout the spine, i.e., not only the neck (19, 20), and have assessed VR technologies with various levels of immersion (19–21). In addition, they may have included patients who experienced specific injuries, i.e., not only non-specific neck pain (19–23), and included study designs other than RCTs (24).

The aim of our study is to assess the effectiveness of therapies based on immersive technologies in the rehabilitation of non-specific neck pain and occurrence of adverse effects. We would also like to determine whether studies on the use of immersive technologies in patients with non-specific neck pain provide sufficient information to allow generalization of the results and their application in clinical practice.

## Methods

### Study selection

A systematic literature search was conducted in July 2023 using Medline (PubMed), Embase (Ovid), Scopus, the Cochrane Database of Systematic Reviews (CDSR), WHO, Pedro, and ClinicalTrials.gov. The search strategy employed a combination of keywords and controlled vocabulary related to immersive technology and non-specific neck pain therapy. The complete search strategies and database-specific retrieval numbers are available in Appendix S1.

Titles and abstracts were independently screened by 2 authors (AZF and DD) and full-text papers were retrieved for all potentially relevant results. Full-text articles were independently reviewed by 2 experts (MP and JSK) against the inclusion criteria and the data from the eligible articles were extracted for presentation by 1 author (JSK) and checked by 2 others working independently (AZF and GF). Where there was a difference between assessments, there was a discussion between assessors to obtain consensus.

The inclusion and exclusion criteria for articles are presented in Table I.

**Table I:** Inclusion and exclusion criteria

Inclusion criteria	Exclusion criteria
- Randomized controlled trials (rcts) - Studies using fully immersive VR technologies (i.e., using a head-mounted display) - Studies written in English - Studies available in full text - Studies with at least 10 participants in the therapeutic group - Adult population older than 18 years old	- Studies related to non-immersive technology - Studies other than rcts - Studies involving children - Studies involving patients with traumatic injuries, significant anatomical changes, or after surgery on the cervical spine and patients with cancer or vestibular conditions

The study protocol was registered in PROSPERO (ID: CRD42023431980; https://www.crd.york.ac.uk/prospero/display_record.php?RecordID=431980). However, the original protocol was altered after registration: 1 author was added (GF) and 1 section regarding the use of immersive technologies in the diagnosis of non-specific neck pain was removed from the purpose of the study (and from the title).

### Data extraction

The following data were extracted from the eligible studies: authors and publication year, included patients (characteristics of participants), study characteristics (PICO at study protocol, selection of patients, healthcare system features, follow-up, statistical analyses), data on impact of VR training on pain, functional limitation and range of motion, and adverse effects. The results (impact of VR training) were presented as the mean with standard deviation for individual groups and the *p*-value (if included in the study).

The generalizability and applicability of the findings of the selected RCTs were assessed by the benchmarking method (BM), in accordance with the CONSORT statement. All descriptive information was extracted by author JK. The accuracy of the extracted data was checked by another author (AZF or GF).

The PRISMA checklist can be found in Appendix S2.

### Risk of bias

The risk of bias was evaluated using the Cochrane Risk of Bias tool (25). This tool consists of 13 questions on potential source of bias. Possible answers were: yes, no, or unsure. The risk of error applies to domains such as: selection bias, performance, attrition, detection, reporting and “other”. Two independent researchers (JSK, GF) evaluated risk of bias of the included studies. In case of inconsistent answers the result was determined by consensus.

## Results

### Data synthesis

**Fig. 1:**
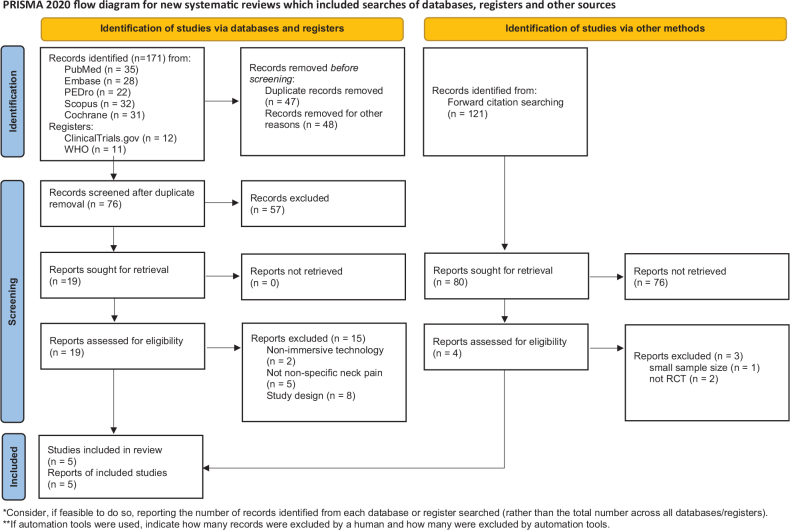
PRISMA flow diagram of the systematic review process. From: Page MJ, McKenzie JE, Bossuyt PM, Boutron I, Hoffmann TC, Mulrow CD, et al. The PRISMA 2020 statement: an updated guideline for reporting systematic reviews. BMJ 2021; 372: n71. doi: 10.1136/bmj.n71.

A total of 171 results were initially exported to EndNote. After duplicates were removed and studies not meeting the criteria were excluded, 76 unique citations remained. After screening according to titles, abstracts and full versions of articles, 4 studies qualified for inclusion. Additionally, the reference lists of included publications were manually searched for further relevant studies (forward citation searching; *n* = 121). In this process, 1 more record was selected. Ultimately, 5 studies meeting the inclusion criteria were included in the review. Five articles published between 2020 and 2023 met the inclusion criteria. The identification process is given in the PRISMA diagram (Fig. 1) (26).

### Characteristics of included studies

The characteristics of these studies are presented in Table II. A total of 203 participants (129 women, 74 men) were enrolled across these studies, with 93 individuals (55 women, 38 men) assigned to VR-based interventions and 110 individuals (74 women, 36 men) assigned to alternative groups.

**Table II:** Study characteristics: PICO at study protocol

Author, year	Inclusion criteria	Exclusion criteria	Intervention characteristics		Outcomes	
			VR group	Control group	Primary outcomes with reference to ICF codes	Secondary outcomes with reference to ICF codes
Battecha, 2023	- Age 18 to 25 years - BMI between 18 and 25 kg/m^2^ - Complaining of non- specific mechanical neck pain	- Neck pain resulting from serious pathology (tumour, rheumatoid arthritis, ankylosing spondylitis, fracture, dislocation, myelopathy, radiculopathy) - Any dermatological conditions - Haemorrhagic blood diseases, - Epilepsy - Long-term use of corticosteroids or receiving any treatment for pain currently - Any previous cervical surgery	VR therapy for all neck movements (included rotation, flexion, side bending, and extension movements – time not given) combined with traditional treatment (stretching, isometric exercises for neck muscles) 3 sessions/week 6 weeks	Traditional treatment only (stretching 3 min and isometric exercises 7 min for neck muscles) 3 sessions/week 6 weeks	CROM: s710 Structure of head and neck region s 7103 Joints of head and neck region s 7104 Muscles of head and neck region b710 Mobility of joint functions VAS: b280 Sensation of pain, b28010 Pain in head and neck NDI: b710 Mobility of joint functions b134 Sleep functions s710 Structure of head and neck region d166 Reading b280 Sensation of pain d510 Washing oneself d540 Dressing d430 Lifting and carrying objects d160 Focusing attention d845 Acquiring, keeping, and terminating a job d920 Recreation and leisure d475 Driving PPT: b280 Sensation of pain, b28010 Pain in head and neck	-
Cetin, 2022	- Age 18 to 65 years - minimum of 6 months of neck pain, - baseline NDI score of at least 20% (10 points), - Neck region as the primary pain area	- Having undergone cervical spine surgery - Rheumatologic, vestibular, neurological, or cardiopulmonary diseases - Receiving interventions including exercise or physical therapy in the previous 6 months - Being pregnant	3 sessions/week for 6 weeks (total of 18 sessions) Each session = 40 min 1. MC exercises 20 min 2. VR for 20 min (5 repetitions for each exercise)	3 sessions/week for 6 weeks (total of 18 sessions) MC (motor control) exercise - each session = 40 min (10 repetitions for each exercise)	JPSE: b260 Proprioceptive function b279 Additional sensory functions, other specified and unspecified CROM: s710 Structure of head and neck region s 7103 Joints of head and neck region s 7104 Muscles of head and neck region b710 Mobility of joint functions	VAS, PPT: b280 Sensation of pain, b28010 Pain in head and neck Muscle strength: b730 Muscle power functions DCFs endurance: b730 Muscle power functions b740 Muscle endurance functions ProFitMap-Necks: s710 Structure of head and neck region b280 Sensation of pain b134 Sleep functions b152 Emotional functions 710 Structure of head and neck region HADS: b152 Emotional functions SF-36: b152 Emotional functions b280 Sensation of pain, b730 Muscle power functions b710 Mobility of joint functions b750–b789 Movement functions d450–d469 Walking and moving d920 Recreation and leisure d230 Carrying out daily routine
Mukherjee, 2020	- Age 30 and above - subacute and chronic cervical spondylosis Without radiculopathy	- Motion sickness - Cervical vertebra fracture - reporting pain > 8 on the (NRS) - Having cervical rib - Diagnosed with mechanical neck pain	Hot pack for 10 min before every session) + 10 min VR session 3 consecutive days	Hot pack for 10 min before every session Conventional treatment (15 min): exercise ROM, scapular retraction, stretching, and cervical core exercise 3 consecutive days	NRS: b280 Sensation of pain, b28010 Pain in head and neck CROM: s710 Structure of head and neck region s 7103 Joints of head and neck region s 7104 Muscles of head and neck region b710 Mobility of joint functions	TKS: b152 Emotional functions d450–d469 Walking and moving d920 Recreation and leisure d230 Carrying out daily routine
Nusser, 2021	- Adults aged 18 years or more - Non-traumatic chronic neck pain (more than 3 months) - No pain medication or muscle relaxants for 24 h before the tests	- Traumatic neck pain - Neck pain originating from whiplash - Cervical fracture/dislocation - Operations in the cervical spine area - Damage to the inner ear - vertebrobasilar insufficiency - Basic neurological diseases - Range of motion of the cervical spine < 10° in flexion, extension, and/or rotation	“Standard rehabilitation programme” + neck-specific sensorimotor training” (NSST) using a VR in individual therapy (6 x 20-min sessions)	1. CG: “Standard rehabilitation programme” 2. SMG: “Standard rehabilitation programme” + over 4 x 30-min group therapy sessions (120 min in total)	NRS: b280 Sensation of pain, b28010 Pain in head and neck CROM: s710 Structure of head and neck region s 7103 Joints of head and neck region s 7104 Muscles of head and neck region b710 Mobility of joint functions NDI: b710 Mobility of joint functions b134 Sleep functions s710 Structure of head and neck region d166 Reading b280 Sensation of pain, d510 Washing oneself d540 Dressing d430 Lifting and carrying objects d160 Focusing attention d845 Acquiring, keeping and terminating a job d920 Recreation and leisure d475 Driving	-
Tejera, 2020	- Age 18 to 65 years - Non-specific chronic neck pain	- Pregnancy - Specific neck pain caused by metastasis, neoplasia, infectious or inflammatory disorders, bone fractures or traumatic precedents with neck injuries - Positive neurological signs or evidence of spinal compression (abnormal diffuse sensitivity, hyperreflexia, or diffuse weakness) - Cervical osteoarthritis - Spondyloarthritis - Neck pain associated with vertigo (vestibular involvement) - Neck pain associated with whiplash injuries - Previous cervical surgeries - Headaches prior to the onset of neck pain and without cervical origin - Inability to provide informed consent	3 series of 10 repetitions of each exercise with 30 s rest between exercises in VR environment 2x/week x 4 weeks 8 session in total	3 series of 10 repetitions of every exercise, with a 30 s rest between exercises 2x/week x 4 weeks 8 session in total	VAS, CPM: b280 Sensation of pain, b28010 Pain in head and neck TS: b280 Sensation of pain, b28010 Pain in head and neck b750–b789 Movement functions	CROM: s710 Structure of head and neck s 7103 Joints of head and neck region s 7104 Muscles of head and neck region b710 Mobility of joint functions NDI: b710 Mobility of joint functions b134 Sleep functions s710 Structure of head and neck region d166 Reading b280 Sensation of pain, d510 Washing oneself d540 Dressing d430 Lifting and carrying objects d160 Focusing attention d845 Acquiring, keeping and terminating a job d920 Recreation and leisure d475 Driving PCS b280 Sensation of pain, b28010 Pain in head and neck b152 Emotional functions TSK: b152 Emotional functions d450–d469 Walking and moving d920 Recreation and leisure d230 Carrying out daily routine FABQ: b152 Emotional functions b750–b789 Movement functions d450–d469 Walking and moving PPT: b280 Sensation of pain, b28010 Pain in head and neck PASS-20: b280 Sensation of pain b28010 Pain in head and neck b152 Emotional functions, b750–b789 Movement functions

CROM: cervical range of motion, ACROM: active CROM, VAS: Visual Analogue Scale, NDI: Neck Disability Index, PPT: pain pressure thresholds, JPSE: joint position sense error, DCFs: deep cervical flexors, ProFitMap-Neck: Profile Fitness Mapping Neck Questionnaire, HADS: Hospital Anxiety and Depression Scale, SF-36: Short Form Health Survey, NRS: Numerical Rating Scale, TSK: Tampa Scale of Kinesiophobia, CPM: conditioned pain modulation, TS: temporal summation, PCS: Pain Catastrophism Scale, FABQ: Fear-Avoidance Beliefs Questionnaire, PASS-20: Pain Anxiety Symptoms Scale.

The most common age criterion for inclusion in the study was an age between 18 and 65 years (27, 28) or no upper age limit (29). One study included people over 30 years of age (30), 1 between 18 and 25 years of age (31). Typically, people with chronic and/or subacute pain defined as non-specific neck pain were included in the study. The exclusion criteria varied greatly (Table II).

In 4 studies, VR therapy was combined with another type of therapy, either exercises (27, 29, 31) or, in 1 group, a hot pack (30). In 1 study, VR therapy was used as monotherapy (28). Control interventions were typically exercise-based therapy described as “conventional treatment”, “standard rehabilitation programme”, “sensorimotor training”, or “motor control exercise”. The characteristics of rehabilitation programmes varied significantly between studies. The time of a single session in the VR environment ranged from 10 to 20 min. In 1 study, the duration of VR exercises was not specified; in another, the duration of the treatment session was given as the number of repetitions and series of exercises. The number of VR exercise sessions ranged from 3 to 18 with different breaks between sessions, and overall duration of the intervention ranged from 3 days to 6 weeks (Table II). All studies assessed the effect of therapy on pain levels, usually with visual analogue scales (VAS; 3 studies), numerical rating scales (NRS; 3 studies) and pain pressure thresholds (PPT; 2 studies), and range of motion. Four studies (27–29, 31) assessed functional limitations related to dysfunctions in the cervical spine, using either the neck disability index (NDI; 3 studies) or ProFitMap-Neck (1 study). Two studies assessed the level of kinesiophobia (28, 30), while the level of depression, quality of life, proprioception, and muscle function were assessed in 1 (27) and psychological indicators in another (28). International Classification of Functioning, Disability and Health (ICF) codes were assigned to individual outcomes (Table II).

Data regarding the selection of patients and features of the healthcare system are presented in Table III. Information regarding recruitment of participants prior to assessment of eligibility was not always clear. Some were recruited from hospital departments (e.g., neurosurgery, rehabilitation), and others through advertisements in social networks or by e-mail. Only 1 study reported the percentage of eligible patients declining participation. In the remaining studies, either no information was given or none of the participants withdrew from participation (27). The order of patient recruitment was documented in flowcharts, except in one study (31). Therapy was usually provided by a physiotherapist, but often no other information was provided (diagnosis, qualification, patient assessment, analysis, etc.) (Table III).

**Table III:** Study characteristics: selection of patients and healthcare system features

Author, year	Patients’ path prior to assessment of eligibility	Reasons for exclusion before randomization	Percentage of eligible patients declining participation documented	Consecutive patient recruitment	Healthcare settings where the data were collected	Staff competence
Battecha, 2023	No information	All eligible participants were randomized	No information	Only information on qualified participants, no information on withdrawal from the study	Faculty of Applied Medical Sciences, physical therapy department (Umm Al-Qura University/Makkah, Saudi Arabia)	Physiotherapist (documentation of the course of exercises and exercise supervision) No other information (diagnosis, qualification, patients assessment, analysis etc.)
Cetin, 2022	Patients recruited from Neurosurgery Department between June 2020 and May 2021, without exercise or physical therapy in the previous 6 months No other information on treatment	- Not meeting inclusion criteria (*n* = 10) - Declined to participate (*n* = 9) - Other reasons (*n* = 2)	14.5% (9 from 61)	Given in flowchart (all the necessary information)	Hacettepe University Hospital’s Neurosurgery Department	Physiotherapist (conducting VR therapy) No other information (diagnosis, qualification, patients assessment, analysis etc.)
Mukherjee, 2020	No information	Not meeting inclusion criteria (*n* = 26)	*n* = 0 (0%)	Given in flowchart (all the necessary information)	Tertiary care hospital in Pune, Maharashtra, India	Diagnosis – orthopaedics specialist Pre- and post-assessment – blinded assessor No other information (diagnosis, qualification, analysis etc.)
Nusser, 2021	Inpatient rehabilitation due to non-traumatic chronic neck pain (more than 3 months) recruited between February 2014 and March 2017	All eligible participants were randomized	*n* = 0 (0%)	Given in flowchart (all the necessary information)	inpatient rehabilitation at the Federseeklinik Bad Buchau (Germany)	Diagnoses: made by patients’ general practitioners, and confirmed by the physician in charge at the rehabilitation hospital Intervention: physiotherapists and certified sports scientists Training instructed by a scientific assistant with a basic education in physiotherapy. Education: orthopaedic specialists and psychologists Assessments: non-blinded scientific assistant
Tejera, 2020	Recruitment through social networks, posters, brochures and emails – no information on patients’ path	All eligible participants were randomized	*n* = 0 (0%)	Given in flowchart (all the necessary information)	Rey Juan Carlos University, CEU San Pablo University	Providing treatments and data collecting (including pain related measurements and psychological variables) – 2 trained physical therapists Statistical analysis – another researcher Writing and reviewing the document – with the help of other assessors

The baseline characteristics of the patients are poorly described in all included articles. Most of the articles failed to provide information on factors that could have influenced the success of the therapy (Table IV). The mean age of the participants varied between studies, i.e., from 21.23 ± 0.83 to 55.81 ± 15 in the VR group and from 21.26 ± 0.79 to 54.81 ± 13 in the alternative groups. One study involved only women, the others included both women and men, but in very different proportions. Baseline pain levels (with VAS or NRS) ranged from 4.29 ± 1.72 to 5.77 ± 1.39 in the VR group and from 3.53 ± 1.84 to (VAS) 5.98 ± 1.93 in the comparison groups. Broader pain characteristics (localization, pain duration, and frequency) were presented in only 1 study (27).

**Table IV:** Study characteristics:baseline characteristics of patients

Author, year	Demographic data		Disorder-specific clinical data		Functioning (D, G, Q)*		Comorbidity		Behavioural factors (S,A, E,O)**		Environmental factors (W, L, M)***		Potential inequity (S, E, D, Et)****	

	VR group	Control group(s)	VR group	Control group(s)	VR group	Control group(s)	VR group	Control group(s)	VR group	Control group(s)	VR group	Control group(s)	VR group	Control group(s)
Battecha, 2023	*n* = 15 F = 15 Age: 21.23 (0.83)	*n* = 15 F = 15 Age: 21.26 (0.79)	pain level (VAS) 4.29 ± 1.72	pain level (VAS) 3.53 (1.84)	D: NDI 10.58(3.84) G:NI Q: NI	D: NDI 10.66(5.47) G: NI Q: NI	NI	NI	S: NI A: NI E: NI O: BMI = 21.33 (2.04)	S: NI A: NI E: NI O: BMI = 20.92 (2.21)	W: students L: NI M: NI	W: students L: NI M: NI	S- NI E –students (physical therapy) D-NI Et- NI	S- NI E –students (physical therapy) D-NI Et- NI
Cetin, 2022	*n* = 17 (F = 12;M = 5) Age: 40.0 (11.88)	*n* = 17 (F = 11;M = 6) Age: 41.94 (10.76)	Pain level (VAS) 5.77 (1.39) Upper neck-10 Lower neck-3 Upper/lower neck-4 Pain duration: 6–12 mth: 6 1–2 y: 4 2–5 y: 5 > 5 y: 2 Pain frequency: ≤1/wk: 1 2–3/wk: 4 > 3/wk: 12	Pain level (VAS) 5.98 (1.93) Upper neck-7 Lower neck-3 Upper/lower neck-7 Pain duration: 6-12 mth: 4 1–2 y: 3 2–5 y: 6 > 5 y: 4 Pain frequency: ≤1/wk:0 2-3/wk:8 > 3/wk:9	D: ProfitMap-Neck (total score): 69.3 (11.3) Q: SF-36 (median with IQR) FF:75.0 (60.0–85.0) PRL: 66.7 (33.3–83.3) ERL:55.0 (55.0–60.0) E: 75.0 (62.5–87.5) EWB:6 8.0 (52.0–74.0) SF: 75.0 (25.0–90.0) P: 45.0 (40.0–62.5) HC: 57.5 (50.0–65.0)	D: ProfitMap-Neck(total score):65.2 (63.49) Q: SF-36 (median with IQR) FF: 75.0 (60.0–90.0) PRL: 33.3 (33.3–66.7) ERL: 45.0 (32.6–55.0) E: 55.0 (38.7–62.5) EWB: 52.0 (46.0–64.0) SF:62.5 (37.5–100.0) P: 45.0 (21.2–65.0) HC: 50.0 (42.5–60.0)	NI	NI	S: NI A: NI E: NI O: BMI = 25.58 (4.35)	S: NI A: NI E: NI O: BMI = 26.31 (4.01)	W: Housewife:3 Workers: 11 Retired: 2 Student: 1 L: NI M: NI	W: Housewife:3 Workers: 10 Retired: 3 Student: 1 L: NI M: NI	S- NI E: PS:2 SS;3 HS:8 U:4 D-NI Et- NI	S- NI E: PS:5 SS;2 HS:8 U:2 D-NI Et- NI
Mukherjee, 2020	*n* = 22 (F = 8;M = 14) Age: 55.81(±15)	*n* = 22 (F = 13;M = 9) Age: 54.81(13)	Pain level (NRS) 5.729 (1.07)	Pain level (NRS) 5.77 (5.05)	D: NI G:NI Q: NI	D: NI G:NI Q: NI	NI	NI	S: NI A: NI E: NI O: NI	S: NI A: NI E: NI O: NI	W:NI L: NI M: NI	W:NI L: NI M: NI	S: NI E: NI D: NI Et: NI	S: NI E: NI D: NI Et: NI
Nusser, 2021	*n* = 17 (F = 9; M = 8) Age 51.2 (8.8)	SMG *n* = 16 (F = 11;M = 5) Age: 53.19 (5.7) CG: *n* = 18 (F = 12;M = 6) Age: 49.8 (8.1)	Non-traumatic chronic neck pain (more than 3 months) Pain level at rest (NRS) 4.9 (2.1) headache at rest: 82%	Non-traumatic chronic neck pain (more than 3 months) Pain level at rest (NRS) SMG: 4.4 (3.1) CG: 4.2 (2.6)	D: NDI 18.7(5.2) G: NI Q: NI	SMG D: NDI 21.5 (6.4) G: NI Q: NI CG D: NDI 18.2 (6.7) G: NI Q: NI	Headache at rest: 82% No other information	Headache at rest SMG: 88% CG: 67% No other information	S: NI A: NI E: NI O: NI	SMG and CG S: NI A: NI E: NI O: NI	W: NI L: NI M: S: 1 M: 9 D: 4 W: 1 U: 2	SMG: W: NI L: NI M: S: 4 M: 10 D: 1 W: 0 U: 1 CG: W: NI L: NI M: S: 3 M: 11 D: 2 W: 1 U:1	S: NI E: NI D: NI Et: NI	SMG and CG S: NI E: NI D: NI Et: NI
Tejera, 2020	*n* = 22 (F = 11;M = 11) Age: 32.72 (11.63)	*n* = 22 (F = 12;M = 10) Age: 26.68 (9.21)	Pain level (VAS) 4.97 (1.88) (4.20–5.74)	Pain level (VAS) 4.27 (1.3) (3.28–4.75)	D: NDI- 13.72 (6.68) (10.76–16.69) G: NI Q: NI	D: NDI - 14.09 (9.32) (9.95–18.22) G: NI Q: NI	NI	NI	S: NI A: NI E: NI O: NI	S: NI A: NI E: NI O: NI	W: NI L: NI M: NI	W: NI L: NI M: NI	S: NI E: NI D: NI Et: NI	S: NI E: NI D: NI Et: NI

* D: disease specific, G: generic, Q: health-related quality of life.

** S: smoking, A: alcohol/substance consumption, E: exercise/physical activity reported, O: obesity (BMI).

*** W: work, L: living conditions, M: marital status.

**** S: socioeconomic status, E: education, D: deprivation, Et: ethnicity.

#: Differences between groups, NI: no information.

PS: Preliminary School, SS: Secondary School, HS: High School, U: University. S: dingle; M: married, D: divorced, W: widowed, U: unknown. CG: control group, SMG: sensorimotor group. SF-36 domains: FF: physical functioning; PRL: role limitations due to physical health, ERL: role limitations due to emotional health; E- energy; EWb: emotional well-being; SF: social functioning; P: pain; HC: health change.

The description of functioning concerned primarily disease-related functioning (3 studies): NDI was used in 2 cases and ProfitMap-Neck in 1. Baseline NDI levels ranged from 10.58 ± 3.84 to 18.7 ± 5.2 in the VR group and from 10.66 ± 5.47 to 21.5 ± 6.4 in the comparison groups. Health-related quality of life was only assessed in 1 study. No other information o functioning was shown in any study.

None of the studies described concomitant health conditions. One only indicated the number of people with headaches. Generally, no information was provided on behavioural factors (2 studies reported BMI – but only as mean with SD) or environmental factors (1 study – occupational status, 1 study – marital status) or potential inequalities (2 studies reported information on education) (Table IV).

Most studies reported the number of patients who completed the entire protocol; however, it was not clear in 1 case. No study performed a crossover (Table V).

**Table V:** Study characteristics: interventions

Author, year	Completed interventions according to protocol among all recruited		Crossover	Co-interventions	
	VR group	Control/other group(s)		VR group	Control group(s)
Battecha, 2023	Not clear “This assessment was limited by the patient’s lessened ability to finish treatment procedures, as patients were experiencing headaches, tired or impaired vision”		No	Traditional exercises	Only traditional exercises
Cetin, 2022	*n* = 17 (from 21)	*n* = 17 (from 20)	No	MC exercises (20 min)	20 min longer MC exercises (additionally to main session)
Mukherjee, 2020	*n* = 22 (from 22)	*n* = 21 (from 22)	No	Hot pack for 10 min before every session	Hot pack for 10 min before every session
Nusser, 2021	*n* = 17 (from 17)	CG *n* = 18(from 20) SMG = 16 (from 18)	No	Standard rehabilitation programme	SMG: standard rehabilitation programme (general sensorimotor training – as a basis) CG: standard rehabilitation programme (only)
Tejera, 2020	*n* = 22 (from 22)	*n* = 22 (from 22)	No	No	No

CG: control group, SMG: sensorimotor group, MC: motor control.

Three of the 5 studies did not assess the validity of the outcome variables. In most cases, the sample size was calculated by statistical software, and in 1 case on feasibility to recruit. Three studies lacked information on the level of power. Follow-up percentage ranged from 82.9% to 100%, with reasons for dropping out or withdrawal (if any) generally provided. The statistical methods were not always selected correctly, with no information given concerning the normality of the distribution, or incorrect tests were selected with regard to the data distribution (Table VI).

**Table VI:** Study characteristics: follow-up, statistical analyses

Author, year	Assessment of validity of outcome variables	Follow-up percentage	Reasons for dropping out/withdrawal		Assessment of power /sample size calculations	Appropriacy of statistical analysis
			VR group	Control group(s)		
Battecha, 2023	No	100%	No one dropped out of the study	No one dropped out of the study	Sample size calculation was made with G* POWER statistical software (version 3.0.10), but no information on power	Only dependent (results before vs past treatment) and independent (comparison of results before therapy between individual groups and after therapy between groups) *t*-test was used. Comments: no information on the normality of the distribution, errors in results
Cetin, 2022	No	82.9% (34 from 41)	Discontinued intervention: - Other health conditions: 2 - Family reasons: 1 - Personal reasons: 1	Lost to follow-up (COVID-19): 1 Discontinued intervention: - Other health conditions: 1 - COVID-19: 1	Sample size calculation was made with G* POWER 3.0 with a power of 80% and a 5% alpha error	Normality of distribution: Kolmogorov–Smirnov/Shapiro–Wilk test Fisher’s χ^2^ test was used for comparing categorical variables between the 2 groups Comparisons of quantitative variables between the groups: *t*-test for normally distributed variables and the Mann–Whitney *U* test for non-normally distributed variables For within-group comparisons with Bonferroni correction Correct statistical analysis
Mukherjee, 2020	No	97.7% (43 from 44)	–	Discontinued intervention (*n* = 1: travelling inconvenience)	Sample size was calculated using the formula for randomized control trials where, Z_α/2_ = 1.96 and Z_þ/2_ = 1.64	Data were explored for normality (all-normally distributed) Within-group analysis for immediate and short‑term: Friedman’s ANOVA test, repeated measures ANOVA test, and Wilcoxon signed‑rank test. Intergroup immediate (Mann–Whitney *U* test) and short‑term effect (unpaired *t*‑test) Despite the normal distribution of the data, non-parametric tests were also used
Nusser, 2021	For patients with neck pain, the NRS: - MDC: 2.1, - MCID: 1.3 NDI: - MDC: 8.4 - MCID: 3.5	92.7% (51 from 55)	No one dropped out of the study	SMG: mistakes in organization (*n* = 2) CG: mistakes in organization *(n* = 2)	Choice of sample size was based on clinical experience and feasibility A sample size of 15–20 patients per group was considered No statistical calculation	Normality of distribution: Shapiro–Wilk test (majority of variables were found to be normally distributed) Within-group differences between pre- and post-intervention: paired 2-tailed *t*-test Basic treatment effects between the 3 groups were examined using a 1-way analysis of variance (ANOVA) For post hoc tests, the Tukey–Kramer test was used Effect sizes of observed between-group changes and its precision: Cohen’s *d* and its 95% confidence interval (95% CI) Not all variables were normally distributed – in such cases, the data should be normalized or non-parametric tests should be used
Tejera, 2020	VAS: validity and reliability confirmed in cited studies MCID: 30 mm CPM: validity and reliability confirmed in cited studies CROM: device verified as reliable for measuring cervical ROM NDI: reliability confirmed in cited studies ICC: 0.50 to 0.98 PCS: a reliable tool with a Cronbach’s α value greater than 0.70 TSK: validity and relia-bility with a Cronbach’s α of 0.79 in a sample of chronic pain FABQ: validity and reliability with a Cronbach’s α of 0.93 PPT: high reliability ICC: 0.91; 95% CI 0.82–0.97) PASS-20: Cronbach’s α of 0.93	100%	No one dropped out of the study	No one dropped out of the study	Sample size calculation performed with G* POWER 3.1.7 with a statistical power of 0.80 and an alpha level of 0.05 Total sample size of 36 patients was estimated Taking into account 15% of the losses, it was necessary to reach a total of 42 patients	Normality of distribution: Shapiro–Wilk test Simple analysis of variance (ANOVA) or mixed variance models (2 × 4) with *post hoc* Bonferroni tests for multiple comparisons were applied Kruskal–Wallis test used to compare the 2 groups at baseline data and at time points Friedman test used to analyde intragroup changes Wilcoxon signed-rank test used for *post hoc* intragroup comparisons Effect size: according to Cohen’s method Correct statistical analysis

MDC: minimal detectable change; MCID: minimum clinically important difference.

CG: control group, SMG: sensorimotor group.

All studies assessed the effect of therapy on pain levels and range of motion; 4 studies (27–29, 31) assessed functional limitations related to dysfunctions in the cervical spine. Two studies assessed the level of kinesiophobia (28, 30); the level of depression, quality of life, proprioception, and muscle function was noted in 1 study (27) and psychological indicators in another (28).

### Effectiveness of VR therapy used for rehabilitation of patients with non-specific neck pain

The effect of therapy on pain levels was assessed in all included studies (Table VII). Three studies used VAS, 2 NRS, and 3 PPT. In 3 cases, both VAS and PPT were used.

**Table VII:** Effect of VR training on pain, functional limitation, and range of motion

Author, year	Pain		Functional limitation		ROM

	Tools	Results	Tools	Results
Battecha, 2023	VAS PPT	Improvement in both groups (the same effect) in VAS (for difference between group after treatment *p* = 0.297) Improvement in both groups in PPT in favour of VRG – statistical significance after treatment for (CG vs VRG): - left side: 4.01 (1.27) vs 5.08 (1.41) (*p* = 0.033) - right side: 4.12 (1.28) vs 4.99 (1.55) (*p* = 0.048)	NDI	Improvement in both groups No differences between groups after treatment (*p* = 0.621)	Improvement in both groups, except extension (in both groups), flexion and flexion to the right in CG No differences between groups after treatment (for all measurements *p* > 0.05)
Cetin, 2022	VAS PPT	VRG and MCG: improvement in PPT – VRG had a significant advantage in PPTs in some localizations (Δ in VRG vs MCG): C_1-2_ left: 3.49 (1.24) vs 2.03 (0.99) (*p* = 0.001) C_1-2_ right: 2.92 (0.89) vs 2.51 ( 0.73) (*p* = 0.03) C_5-6_ left: 2.04 (1.01) vs 0.96 (0.88) (*p* = 0.002) C_5-6_ right: 2.24 (1.36) vs 1.32 (0.81) (*p* = 0.02) No between-group differences in the deltas of VAS (*p* = 0.07)	ProFitMap-Neck	VRG: improvement in (symptom frequency and total index) (*p* < 0.001) MCG: improvement in symptom frequency, functional limitation, and total index (*p* < 0.001) No significant differences between the groups, except for functional limitation index – VRG had a greater improvement (Δ in VRG vs MCG): 14.64 (8.93) vs 6.36 (13.68) (*p* = 0.04)	VRG: improvement (*p* < 0.01) MCG: improvement (except lateral flexions) No differences between groups (*p* > 0.05)
Mukherjee, 2020	NRS	Improvement in both groups (*p* < 0.01 at all analysed time points) Significant difference in the intergroup analysis (pre–post VRG vs pre-post CG): - immediately: 5.77 (5.05) - 4.65 (1.08) vs 5.72 (1.07) -3.94 (1.67) (*p* = 0.02) - in the short term: 5.77 (5.05)- 1.5 (0.80) vs 5.72 (1.07) - 2.71 (1.45) (*p* = 0.00)	-	-	Improvement in both groups VRG – better improvement in the short term (pre-post VRG vs pre-post CG): - L rotation: 48.90 (11.30)– 65.77 (9.31) vs 53 (14.13)– 58.95 (11.28) (*p* = 0.04) - R rotation: 52.54 (11.55)–66.18 (6.95) vs 52.04 (12.33)– 58.42 (10.27) (*p* = 0.01) - L lateral flexion: 31.5 (8.86)–51.04 (9.06) vs 38.36 (13.81)–45.90 (14.68) (*p* = 0.00) - R lateral flexion: 32.40 (12.87)– 49.86 (10.39) vs 37 (11.75)– 49.19 (11.80) (*p* = 0.01)
Nusser, 2021	NRS	SMG: improvement in headache at rest (*p* < 0.01) VRG: improvement in all aspects of pain (at rest *p* < 0.01, during motion *p* < 0.05, headache at rest *p* < 0.01, headache during motion *p* < 0.01) For headache better result for VR vs CG (pre–post VRG vs pre–post CG) - at rest: 3.8 (3.0)–0.4 (0.7) vs 2.7 (2.4)–2.0 (2.1) (*p* < 0.008) - during motion 4.7 (3.4)–1.1 (1.2) vs 2.7 (2.9)–2.3 (2.6) (*p* < 0.023)	NDI	Improvement in both groups (*p* < 0.01), with no advantage in any one group	Improvement in VRG (for flexion *p* < 0.05, extension *p* < 0.001, and left rotation *p* < 0.05) Compared with CG for increase in flexion and extension, the differences between groups were statistically significant in favour of the VRG (pre-post VRG vs pre-post CG): - flexion: 40.9 (14.6)–48.5 (13.3) vs 45.8 (12.9)–42.9 (12.6) (*p* < 0.05) - extension: 35.4 (12.8)–44.6 (12.9) vs 43.1 (13.3)–39.8 (14.7) (*p* < 0.01) For extension VRG vs SMG in favour of the VRG (pre-post VRG vs pre-post SMG - extension: 35.4(12.8)- 44.6(12.9) vs 39.1(12.7) – 37.7(15.1) (*p* < 0.05)
Tejera, 2020	VAS, CPM (with PPT)	Improvement in both groups: - VRG: post-treatment (*p* = 0.01), 1 month follow-up (*p* < 0.01) and 3 month follow-up (*p* < 0.01) -CG: post-treatment (*p* < 0.01), 1 month follow-up (*p* < 0.01) and 3 months follow-up (*p* < 0.01) No group*time interaction	NDI	Improvement in both groups (*p* < 0.01) No group*time interaction	Significant effects - for time factor (*p* < 0.05) but not for the group*time interaction (*p* > 0.05) for rotation - not for time factor (*p* > 0.05) and not in group*time interaction (*p* > 0.05) for flexor-extension and lateral-flexion ROM

CG: control group; VRG: virtual reality group, MC: motor control group, SMG: sensorimotor group, ROM: range of motion, NDI: Neck Disability Index, VAS: Visual Analogue Scale, PPT: pain pressure thresholds, NRS: Numerical Rating Scale.

In most cases, the VR and control groups demonstrated improvement in pain. When the pain level was measured using the VAS, neither therapy demonstrated any advantage (27, 28, 31). When NRS was used, the results were more favourable in the VR group (29, 30), but in 1 study (for immediate effect) favourable for control (30). Where PPT was used, either the VR group had a significant advantage in some localizations (27), or no advantage was recorded for either group (28).

With regard to functioning, better NDI scores were achieved in all study groups, with no clear advantage in any of them. No significant differences were found between groups in the study (27) that used ProFitMap-Neck; however, a greater improvement in functional limitation index was observed in the VR group (Table VII).

With a few exceptions, improved range of motion was noted in the cervical spine after therapy. In most cases, no difference was noted between the groups; the only differences were observed in favour of the VR group (Table VII).

### Other results

The therapy generally improved kinesiophobia; However, no differences between groups were noted in one study (30) (*p* = 0.25), and significant differences were revealed for group*time interaction in another (28) (F = 3.89, *p* = 0.01, η_p_^2^ = 0.08): *post hoc* differences were observed in favour of the VR group at 3 months (*p* < 0.05, *d* = 0.65).

Individual studies also assessed muscle function, quality of life, proprioception, and psychological distress. No differences in muscle strength, endurance, hospital anxiety and depression scale (HADS), or short form-36 (SF-36) were found between groups following therapy (*p* > 0.05); however, the VR group had a significant advantage in joint position sense error (JPSE) (Δ in VRG vs MCG); flexion (–2.81) ± 1.82 vs (–1.16) ± 1.17 (*p* = 0.04); extension (–2.80) ± 1.84 vs (–1.52) ± 1.16 (*p* = 0.02); right lateral flexion (–3.53) ± 1.35 vs (–2.62) ± 1.36 (*p* = 0.03); left lateral flexion (–3.95) ± 1.03 vs (–2.95) ± 4.27 (*p* = 0.04); right rotation (–4.07) ± 2.99 vs (–1.15) ± 1.0 (*p* = 0.001); left rotation (–2.81) ± (–1.64) vs (–1.17) ± 1.24 (*p* = 0.002).

No group*time interaction was noted for pain catastrophism (PCS), fear-avoidance beliefs (FABQ), or anxiety (PASS-20).

### Adverse effects

The adverse effects associated with VR were not described sufficiently (Table VIII). In the studies presented, either none of the patients complained about any adverse events (27), they were not reported (28, 30), or they were described quite imprecisely. In the Battecha et al. study (31), only information that patients experienced headaches, tiredness, or impaired vision was reported. In Nusser et al. (29), some patients complained about the weight of the helmet, but no information was provided on the number of complaints. No other negative side effects were reported regarding the VR device or in general.

**Table VIII:** Adverse effects associated with VR therapy

Author, year	Adverse effects	Comments
Battecha, 2023	Headaches, tired or impaired vision	No information on the number of people and the severity of the symptoms (information from study limitation section)
Cetin, 2022	No adverse effects were observed in either group	–
Mukherjee, 2020	Not reported	Participants’ reporting of motion sickness was excluded from the study
Nusser, 2021	Some patients found the weight of the helmet unpleasant No other negative side effects were reported regarding the VR device or in general	No information on the number of complaints (“some patients”)
Tejera, 2020	Not reported	Motion sickness produced by virtual reality headsets was not taken into account (information from study limitation section)

### Risk of bias

Risk of bias is presented in Table IX. The most problematic points were those regarding performance domains or other risk of bias and the least problematic were those concerning attrition. In all included studies, other sources of potential bias were also identified.

**Table IX:** Risk of bias of the included studies

Bias domain	Source of bias	Battecha et al. (2023)	Cetin et al. (2022)	Mukherjee et al. (2020)	Nusser et al. (2021)	Tejera et al. (2020)
Selection	(1) Was the method of randomization adequate?	+	+	?	+	+
Selection	(2) Was the treatment allocation concealed?	+	?	-	?	+
Performance	(3) Was the patient blinded to the intervention?	-	-	-	-	-
Performance	(4) Was the care provider blinded to the intervention?	-	-	-	-	-
Detection	(5) Was the outcome assessor blinded to the intervention?	?	?	+	?	+
Attrition	(6) Was the dropout rate described and acceptable?	-	+	+	+	+
Attrition	(7) Were all randomized participants analysed in the group to which they were allocated?	+	+	+	+	+
Reporting	(8) Are reports of the study free of suggestion of selective outcome reporting?	+	+	+	+	+
Selection	(9) Were the groups similar at baseline regarding the most important prognostic indicators?	+	+	?	?	+
Performance	(10) Were co-interventions avoided or similar?	+	+	+	+	+
Performance	(11) Was the compliance acceptable in all groups?	-	+	-	-	+
Detection	(12) Was the timing of the outcome assessment similar in all groups?	+	+	+	+	+
Other	(13) Are other sources of potential bias unlikely?	-	-	-	-	-

## DISCUSSION

### Effectiveness of immersive technologies in the rehabilitation of participants with non-specific neck pain

Despite the increasing use of VR in rehabilitation, the body of research is insufficient to allow the use of standardized therapy protocols in specific clinical groups (32). Therefore, the primary aim of this review was restricted to evaluating the effectiveness of immersive technologies in the rehabilitation of participants with *non-specific neck pain*. In the reviewed papers, the most frequently analysed indicators were pain and range of motion in the head and neck, as well as functioning related to neck pain. In most cases, the therapies led to a reduction in pain levels, both in the VR and in the control/alternative therapy groups. In some cases, neither form of therapy demonstrated any advantage in terms of effectiveness (27, 28, 31); however, VR-based therapy demonstrated a more beneficial effect in others (29, 30). Even so, 1 study achieved a better immediate effect in the control group, despite the authors’ interpretation (30). In general, hence, VR therapy could be considered promising.

The outcome measures associated with the quality of everyday functioning related to neck pain (NDI and ProFitMap-Neck) also improved as a result of the applied therapies. Similar improvements were noted for all study groups with 1 exception, where the VR group demonstrated a greater improvement in the ProFitMap-Neck (functional limitation index) domains (27).

In most cases, range of movement improved with therapy; however, single directions of movement did not improve in some studies. Even so, in 3 of the 5 studies, these changes did not indicate an advantage of either therapy (27, 28, 31); in the other 2, the VR-based therapy yielded greater improvements in relation to rotation and lateral flexion (30) or flexion and extension (29).

As with other outcome measures, therapies resulted in improvement in the fear of movement (kinesiophobia) (28, 30). However, no clear advantage in the effectiveness of any of the therapies was obtained, except for better long-term effects after VR therapy in 1 of the studies (28). Some improvement was noted in muscle function, quality of life, proprioception, and emotional function with therapy, but usually neither therapy demonstrated any advantage. However, VR therapy demonstrated greater improvements in JPSE, i.e., an expression of proprioception function, with better results observed at each assessed point (27).

The effectiveness of VR therapy may result from greater engagement in the therapy process. Such training allows interaction with a virtual environment, gives a feeling of “being physically present”, which is beneficial during rehabilitation (32, 33). However, none of the studies included in the review analysed the degree of acceptability of the therapy for the patient, involvement, or motivation to start or continue therapy (VR vs another type of therapy). VR therapy can be treated as another form of exercise, where the therapeutic agent is movement/exercise, but delivered in a more acceptable form, and not as another form of therapy. Exercise is the preferred non-pharmacological form of evidence-based therapy for the treatment of patients with neck pain, constituting a fundamental element of therapy guidelines for this group of patients (4). It is also worth noting that, with minor exceptions, VR-based therapy was used together with exercise-based intervention. It is therefore very likely that exercises contributed to the success of the therapy. However, Tejera et al. (28) did not use any co-interventions, but no group-time interaction was reported. Also, Mukherjee et al. (30) used a hot pack rather than exercise as the co-intervention; in this study, the VR group performed better in terms of both pain assessment and range of motion.

### Heterogeneity of the studies

Although VR-based therapy achieved promising results, questions arise as to whether these results can be generalized and transferred to clinical practice. The reviewed articles demonstrate considerable heterogeneity with regard to patient groups and therapy characteristics (time of a single unit, observation time, different methods, different co-interventions). Studies typically involved women and men, in different proportions, but 1 study included only women (31). Significant age differences were noted between them, with the mean age ranging from 21.2 to 55.8 years in VR group. In addition, the intervention ranged from 3 (10-min sessions, on 3 consecutive days) (30) to 18 sessions (3 x 40-min sessions/week for 6 weeks) with each session consisting of 20 min of VR plus 20 min of other exercises (27). In 1 of the studies, the therapy parameters differed between the VR group (6 times for 20 min) and control groups (4 times for 30 min) (29).

### Applicability of findings to clinical practice

As the primary goal of a systematic review is to help clinicians select appropriate therapeutic methods for clinical practice, the present review includes data that may be important when making clinical decisions. A comprehensive assessment should include not only information on features directly related to the condition, but other important elements that may affect the effectiveness of therapy. According to the benchmarking review method, such assessments should include 5 categories (selection, baseline characteristics, intervention factors, outcome assessments, and statistical) with several subcategories (34). Even reviews published in leading journals do not always take into account the exact characteristics of the included articles; this omission can obscure the similarity between the included studies, the patients to which these results could be generalized, and their value (18, 35).

The benchmarking method first requires information on patient selection, including inclusion and exclusion criteria. The most common criteria for inclusion in a study were age (as described above) and the presence of chronic or chronic/subacute non-specific neck pain. However, 1 study also included BMI (31), and another included a baseline NDI score of at least 20% (10 points) and the neck as the primary pain area (27). The exclusion criteria were described in more detail; they mostly included specific pain resulting from serious pathology, including injuries (27–31), currently receiving any treatment for pain (27, 29, 31), pregnancy (27, 28), cervical surgery (27–29, 31), vertebrobasilar insufficiency (28, 29), or neurological conditions (27–31). In addition, individual studies also excluded participants based on the presence of a cervical rib, motion sickness, severe pain (> 8 on the NRS) (30), haemorrhagic conditions, epilepsy, any dermatological conditions (31), damage to the inner ear, range of motion of the cervical spine < 10° in flexion, extension, and/or rotation (29), inability to provide informed consent, or headaches prior to the onset of neck pain and without cervical origin (28).

Most of the studies did not describe the recruitment process in detail. Although2 mentioned the site or method of patient recruitment, e.g., patients who underwent inpatient rehabilitation at a specific unit (29) or the Neurosurgery Department (27), the diagnostic or therapeutic path before participation was not given. Four studies report consecutive recruitment, as indicated in a flowchart. However, in 3 of the 5 studies, none of the patients left before randomization, suggesting that all met the inclusion criteria, and only 1 study reported that some patients declined participation (14.5%) before randomization. In most cases, the research was conducted in academic units or medical units operating at universities. With some exceptions, for example, an orthopaedic specialist (30), or the physician-in-charge at the rehabilitation hospital (29), no indication was given of the competences of the individual providing the diagnosis, patient assessment, or analysis. Therapy and exercise programmes were conducted by physiotherapists, although 1 study used certified sports scientists with training given by a scientific assistant with a basic training in physiotherapy (29). In 1 case, a physical therapist also took measurements of pain-related and psychological variables (28).

The benchmarking method also examines the validity and completeness of baseline data. Although the studied groups should be characterized by similar baseline values (36), this is not always the case. It was not always clear in the examined studies whether the groups were similar at baseline, even in respect of the most important prognostic indicators, such as age, sex, functional status, or clinical parameters (29, 30). Even if these data were presented, it was not always known whether they differed significantly. Apart from the pain level, most studies lacked specific clinical data; however, pain localization, frequency, and duration were reported in Cetin et al. (27), and additional information concerning pain at rest and in motion, as well as headache, was given by Nusser et al. (29). Only 1 study described concomitant conditions, such as headaches (29), and 2 indicated behavioural factors, such as information on mean BMI (27, 31). One study gave information on quality of life, based on the SF-36 (27), another 2 reported environmental factors (1 described occupational status and the other marital status) and another 2 indicated potential inequalities (namely information on education).

Sample size calculations were generally provided, with a statistical power estimate of 80%, which is the standard for adequacy. In 1 case, the choice of sample size was based on clinical experience and feasibility (29), but no statistical calculations were presented. Three of the 5 studies reported reasons for dropout or withdrawal, and in the remaining 2 studies, 100% of participants completed the study. Three out of the 5 studies did not assess the validity of the outcome variables. In some of the articles, the statistical analysis was not performed correctly, e.g., either no information was given regarding the normality of the distribution and only parametric tests were used, or non-parametric tests were used despite the data being normally distributed.

### Adverse effects

The second goal of this study was to determine whether the use of VR technologies is associated with adverse effects in patients with non-specific neck pain. It was hypothesized that VR therapy may elicit dizziness, vertigo, blurred vision, nausea, and difficulty focusing, known collectively as *cybersickness* or *VR sickness*. In 1 review, the mean dropout rate reported across 46 experiments due to VR sickness was 15.6% (37). We also hypothesised that any additional weight placed on the head (glasses, head-mounted display) could potentially worsen the symptoms of neck pain. In the reviewed papers, either none of the patients complained about any adverse events (27), they were not reported (28, 30), or they were described quite imprecisely. For example, in the Battecha et al. study (31), the *Limitations* section states that some patients were unable to complete treatment procedures due to headaches, fatigue, or visual disturbances. No other information regarding the severity of symptoms or the number of cases was provided.

In Nusser et al. (29), no reports of symptoms typical of VR-associated sickness were given, but some complaints about the weight of the helmet were noted; however, the number was not provided. In addition, the *Methods* section notes that “Rest breaks of approximately 3 min were given between tasks, and extended if any side-effect (e.g. motion sickness, nausea, or headaches) was reported. Patients only continued if side-effect effects subsided, otherwise they would stop the training session.” This suggests that this part of the study was insufficiently developed.

The authors of some studies tried to eliminate the factors causing cybersickness. In 1 study (30), patients with motion sickness were excluded at the recruitment stage (38). Another study (27) employed an application that was designed to avoid VR sickness. None of the studies included in the review prepared a protocol for recording adverse events at the study planning stage and the occurrence of adverse events was not accurately reported. Further research is necessary to assess the prevalence and risk of these events, with the cases recorded using appropriate protocols, particularly in patients with cervical spine pain.

### Strength and limitations

The strengths of this review are that we included only randomized controlled trials and limited our studies to those related to nonspecific neck pain, in contrast to other reviews on this topic, which analysed studies related to different parts of the spine or did not exclude studies that also included specific neck pain, e.g., neck pain following injuries. Our goal was to limit the heterogeneity of the analysed studies. Despite our efforts, the studies were characterized by considerable heterogeneity in terms of both patient characteristics (primarily the very diverse age of the study participants) and the characteristics of the intervention programmes used.

Moreover, due to poor reporting of adverse events, we were unable to adequately describe the potential risks associated with the use of VR devices in the rehabilitation of patients with nonspecific neck pain. This indicates the need to include these extremely important issues in future studies, especially among patients with dysfunctions in this area of the body. Despite the increasingly developing rehabilitation sector using immersive devices, the applicability of evidence remains limited.

### Conclusion

Although evidence suggests that VR-based therapy may have benefits in the rehabilitation of patients with non-specific neck pain, these findings should be treated with caution. A great many significant basic data are still missing, and the existing body of studies is characterized by considerable heterogeneity, in terms of both the interventions themselves and patient characteristics. Most of the studies analysed lacked sufficient information regarding patient selection, disease-specific data, functional characteristics, and comorbidities, as well as environmental, behavioural, and equity-related factors. Furthermore, insufficient data on the safety of therapy and adverse events exist to draw any conclusions regarding safety.

There is a pressing need for more comprehensive RCTs investigating VR-based therapy, employing standard benchmarking methods for better reporting. Furthermore, such research should follow appropriate protocols for recording adverse events to ensure that VR therapy does not pose any risk to patients.

## Supplementary Material

EFFECTIVENESS AND ADVERSE EFFECTS OF IMMERSIVE TECHNOLOGIES USED FOR REHABILITATION OF PATIENTS WITH NON-SPECIFIC NECK PAIN: A SYSTEMATIC REVIEW
