# Supplementary material for: EFFECTIVENESS AND ADVERSE EFFECTS OF IMMERSIVE TECHNOLOGIES USED FOR REHABILITATION OF PATIENTS WITH NON-SPECIFIC NECK PAIN: A SYSTEMATIC REVIEW
Source: J Rehabil Med. 2025 Jan 31;57:42108. doi: 10.2340/jrm.v57.42108 (PMC11811534; doi:10.2340/jrm.v57.42108)
Supplement: EFFECTIVENESS AND ADVERSE EFFECTS OF IMMERSIVE TECHNOLOGIES USED FOR REHABILITATION OF PATIENTS WITH NON-SPECIFIC NECK PAIN: A SYSTEMATIC REVIEW [file JRM-57-42108-s1.pdf]

Supplementary material has been published as submitted. It has not been copyedited, or typeset by Journal of Rehabilitation Medicine

Appendix S1.

Search strategy:

PubMed

Nr	Kwerenda
#1	**(„neck pain"[mh] OR „Neck Pain*"[tiab] OR „Neck Ache*" [tiab] OR Cervicalgia*[tiab] OR Cervicodynia*[tiab] OR Neckache*[tiab] OR „Cervical Pain*"[tiab] OR „Posterior Cervical Pain*"[tiab] OR „Posterior Neck Pain*" [tiab] OR „Anterior Cervical Pain*"[tiab] OR „Anterior Neck Pain*"[tiab])**
#2	**("virtual reality"[MeSH Terms] OR ("virtual"[tiab] AND "reality"[tiab]) OR "virtual reality"[tiab] OR Machine Learning[mh] OR "machine learning"[tiab] OR "transfer learning"[tiab] OR Virtual Reality Exposure Therapy[mh] OR "Virtual Reality Therapy"[tiab] OR Artificial Intelligence[mh] OR artificial intelligence[tiab] OR "Computational Intelligence"[tiab] OR "Machine Intelligence"[tiab] OR "Computer Reasoning"[tiab] OR "Computer Vision Systems"[tiab] OR "Computer Vision System"[tiab] OR „immersive technolog*"[tiab])**
#3	**(clinical[tiab] AND trial[tiab]) OR "clinical trials as topic"[mesh] OR "clinical trial"[pt] OR random*[tiab] OR "random allocation"[mesh] OR "therapeutic use"[sh])**
#4	#1 AND #2 AND #3

Embase

Nr	Kwerenda
#1	1 exp neck pain/ or (Neck adj2 Pain$1).ti,ab,kf. or (Neck adj2 Ache$1).ti,ab,kf. or (Cervicalgia$1 or Cervicodynia$1 or Neckache$1).ti,ab,kf. or (Cervical adj2 Pain$1).ti,ab,kf. or (Posterior adj2 Cervical adj2 Pain$1).ti,ab,kf. or (Posterior adj2 Neck adj2 Pain$1).ti,ab,kf. or (Anterior adj2 Cervical adj2 Pain$1).ti,ab,kf. or (Anterior adj2 Neck Pain$1).ti,ab,kf.
#2	(clinical adj2 trial).ti,ab,kf. or exp clinical trial/ or random*.ti,ab,kf. or exp randomization/ or randomisation.ti,ab,kf. or (random adj2 allocation).ti,ab,kf.
#3	exp exergaming/ or (exergaming or exergam$3).ti,ab,kf. or exp virtual reality/ or ((virtual adj2 realit$3) or VR or (computer adj2 simulated adj2 environment) or (augmented adj2 realit$3) or (simulated adj2 realit$3) or (simulated adj2 environment) or (virtual adj2 simulation) or (virtual adj2 technolog$3) or (simulation adj2 technolog$3) or (video adj2 game$1) or (virtual adj2 environment) or (virtual adj2 therap$3) or (virtual adj2 exercise$1) or (virtual adj2 rehabilitation) or (immersive adj2 technolog$3)).ti,ab,kf.
#4	1 and 2 and 3
#5	4 not conference*.pt.

Scopus

Nr	Kwerenda
#1	(TITLE-ABS-KEY ({Neck Pain} OR {Neck Ache} OR cervicalgia OR cervicodynia OR neckache OR {Cervical Pain} OR {Posterior Cervical Pain} OR {Posterior Neck Pain} OR {Anterior Cervical Pain} OR {Anterior Neck Pain}) AND TITLE-ABS-KEY (exergaming OR "virtual realit*" OR vr OR {computer simulated environment} OR "augmented realit*" OR "simulated realit*" OR {simulated environment} OR {virtual simulation} OR "virtual technolog*" OR "simulation technolog*" OR "video game*" OR {virtual environment} OR "virtual therap*" OR "virtual exercise*" OR {virtual rehabilitation} OR "immersive technolog*") AND TITLE-ABS-KEY ("clinical trial" OR random* OR randomi?ation OR {random allocation}))

Cochrane

Cochrane Database of Systematic Review (CDRS)

Nr	Kwerenda
#1	(exergaming OR "virtual reality" OR vr OR computer simulated environment OR "augmented reality" OR "simulated reality" OR simulated environment OR virtual simulation OR "virtual technology" OR "simulation technology" OR "video games" OR virtual environment OR "virtual therapy" OR "virtual exercises" OR virtual rehabilitation OR "immersive technology"):ti,ab,kw
#2	("neck pain"):ti,ab,kw
#3	#1 AND #2

clinicaltrials.gov

Nr	Kwerenda
#1	*Condition*: neck
#2	*Other terms:* virtual reality

WHO

World Health Organizatiom (WHO) International Clinical Trials Registry Platform (ICTRP)

ICTRP Search Portal Advanced Search (who.int)

Nr	Kwerenda
#1	*Intervention*: exergaming OR "virtual reality" OR vr OR „computer simulated environment” OR "augmented reality" OR "simulated reality" OR simulated environment OR „virtual simulation” OR "virtual technology" OR "simulation technology" OR "video game" OR „virtual environment” OR "virtual therapy" OR "virtual exercise" OR „virtual rehabilitation” OR "immersive technology"

PEDro

Nr	Kwerenda
#1	*Abstract & title:* virtual reality
#2	*Body part*: head neck

Appendix S2. PRISMA – checklist

Section and Topic	Item #	Checklist item	Location where item is reported
**TITLE**			
Title	1	Identify the report as a systematic review.	P. 1
**ABSTRACT**			
Abstract	2	See the PRISMA 2020 for Abstracts checklist.	
**INTRODUCTION**			
Rationale	3	Describe the rationale for the review in the context of existing knowledge.	P. 4
Objectives	4	Provide an explicit statement of the objective(s) or question(s) the review addresses.	P. 4
**METHODS**			
Eligibility criteria	5	Specify the inclusion and exclusion criteria for the review and how studies were grouped for the syntheses.	P. 5
Information sources	6	Specify all databases, registers, websites, organisations, reference lists and other sources searched or consulted to identify studies. Specify the date when each source was last searched or consulted.	P. 5
Search strategy	7	Present the full search strategies for all databases, registers and websites, including any filters and limits used.	App. 1
Selection process	8	Specify the methods used to decide whether a study met the inclusion criteria of the review, including how many reviewers screened each record and each report retrieved, whether they worked independently, and if applicable, details of automation tools used in the process.	P. 5
Data collection process	9	Specify the methods used to collect data from reports, including how many reviewers collected data from each report, whether they worked independently, any processes for obtaining or confirming data from study investigators, and if applicable, details of automation tools used in the process.	P. 5
Data items	10a	List and define all outcomes for which data were sought. Specify whether all results that were compatible with each outcome domain in each study were sought (e.g. for all measures, time points, analyses), and if not, the methods used to decide which results to collect.	P. 5
	10b	List and define all other variables for which data were sought (e.g. participant and intervention characteristics, funding sources). Describe any assumptions made about any missing or unclear information.	P. 5
Study risk of bias assessment	11	Specify the methods used to assess risk of bias in the included studies, including details of the tool(s) used, how many reviewers assessed each study and whether they worked independently, and if applicable, details of automation tools used in the process.	P. 6
Effect measures	12	Specify for each outcome the effect measure(s) (e.g. risk ratio, mean difference) used in the synthesis or presentation of results.	P. 5
Synthesis methods	13a	Describe the processes used to decide which studies were eligible for each synthesis (e.g. tabulating the study intervention characteristics and comparing against the planned groups for each synthesis (item #5)).	no
	13b	Describe any methods required to prepare the data for presentation or synthesis, such as handling of missing summary statistics, or data conversions.	no
	13c	Describe any methods used to tabulate or visually display results of individual studies and syntheses.	no

Section and Topic	Item #	Checklist item	Location where item is reported
	13d	Describe any methods used to synthesize results and provide a rationale for the choice(s). If meta-analysis was performed, describe the model(s), method(s) to identify the presence and extent of statistical heterogeneity, and software package(s) used.	no
	13e	Describe any methods used to explore possible causes of heterogeneity among study results (e.g. subgroup analysis, meta-regression).	no
	13f	Describe any sensitivity analyses conducted to assess robustness of the synthesized results.	no
Reporting bias assessment	14	Describe any methods used to assess risk of bias due to missing results in a synthesis (arising from reporting biases).	P. 6
Certainty assessment	15	Describe any methods used to assess certainty (or confidence) in the body of evidence for an outcome.	no
**RESULTS**			
Study selection	16a	Describe the results of the search and selection process, from the number of records identified in the search to the number of studies included in the review, ideally using a flow diagram.	Fig. 1
	16b	Cite studies that might appear to meet the inclusion criteria, but which were excluded, and explain why they were excluded.	Fig. 1
Study characteristics	17	Cite each included study and present its characteristics.	Tab. 1-6
Risk of bias in studies	18	Present assessments of risk of bias for each included study.	Tab. 7
Results of individual studies	19	For all outcomes, present, for each study: (a) summary statistics for each group (where appropriate) and (b) an effect estimate and its precision (e.g. confidence/credible interval), ideally using structured tables or plots.	no
Results of syntheses	20a	For each synthesis, briefly summarise the characteristics and risk of bias among contributing studies.	Tab. 5
	20b	Present results of all statistical syntheses conducted. If meta-analysis was done, present for each the summary estimate and its precision (e.g. confidence/credible interval) and measures of statistical heterogeneity. If comparing groups, describe the direction of the effect.	Tab. 6
	20c	Present results of all investigations of possible causes of heterogeneity among study results.	Tab. 1-6
	20d	Present results of all sensitivity analyses conducted to assess the robustness of the synthesized results.	no
Reporting biases	21	Present assessments of risk of bias due to missing results (arising from reporting biases) for each synthesis assessed.	Tab. 2-6
Certainty of evidence	22	Present assessments of certainty (or confidence) in the body of evidence for each outcome assessed.	No
**DISCUSSION**			
Discussion	23a	Provide a general interpretation of the results in the context of other evidence.	P. 8, 9
	23b	Discuss any limitations of the evidence included in the review.	P. 9-11
	23c	Discuss any limitations of the review processes used.	P. 9-11
	23d	Discuss implications of the results for practice, policy, and future research.	P. 10-12

Section and Topic	Item #	Checklist item	Location where item is reported
**OTHER INFORMATION**			
Registration and protocol	24a	Provide registration information for the review, including register name and registration number, or state that the review was not registered.	P. 5
	24b	Indicate where the review protocol can be accessed, or state that a protocol was not prepared.	P. 5
	24c	Describe and explain any amendments to information provided at registration or in the protocol.	P. 5
Support	25	Describe sources of financial or non-financial support for the review, and the role of the funders or sponsors in the review.	P. 12
Competing interests	26	Declare any competing interests of review authors.	P. 12
Availability of data, code and other materials	27	Report which of the following are publicly available and where they can be found: template data collection forms; data extracted from included studies; data used for all analyses; analytic code; any other materials used in the review.	P. 12

From: Page MJ, McKenzie JE, Bossuyt PM, Boutron I, Hoffmann TC, Mulrow CD, et al. The PRISMA 2020 statement: an updated guideline for reporting systematic reviews. BMJ 2021;372:n71. doi: 10.1136/bmj.n71
